# Neurodevelopmental outcome in preterm infants with intraventricular hemorrhages: the potential of quantitative brainstem MRI

**DOI:** 10.1093/cercor/bhae189

**Published:** 2024-05-07

**Authors:** Patric Kienast, Victor Schmidbauer, Mehmet Salih Yildirim, Selina Seeliger, Marlene Stuempflen, Julia Elis, Vito Giordano, Renate Fuiko, Monika Olischar, Klemens Vierlinger, Christa Noehammer, Angelika Berger, Daniela Prayer, Gregor Kasprian, Katharina Goeral

**Affiliations:** Department of Biomedical Imaging and Image-Guided Therapy, Division of Neuroradiology and Musculoskeletal Radiology, Medical University of Vienna, Währinger Gürtel 18-20, 1090 Vienna, Austria; Department of Biomedical Imaging and Image-Guided Therapy, Division of Neuroradiology and Musculoskeletal Radiology, Medical University of Vienna, Währinger Gürtel 18-20, 1090 Vienna, Austria; Department of Biomedical Imaging and Image-Guided Therapy, Division of Neuroradiology and Musculoskeletal Radiology, Medical University of Vienna, Währinger Gürtel 18-20, 1090 Vienna, Austria; Department of Biomedical Imaging and Image-Guided Therapy, Division of Neuroradiology and Musculoskeletal Radiology, Medical University of Vienna, Währinger Gürtel 18-20, 1090 Vienna, Austria; Department of Biomedical Imaging and Image-Guided Therapy, Division of Neuroradiology and Musculoskeletal Radiology, Medical University of Vienna, Währinger Gürtel 18-20, 1090 Vienna, Austria; Department of Pediatrics and Adolescent Medicine, Division of Neonatology, Pediatric Intensive Care and Neuropediatrics, Comprehensive Center for Pediatrics, Medical University of Vienna, Währinger Gürtel 18-20, 1090 Vienna, Austria; Department of Pediatrics and Adolescent Medicine, Division of Neonatology, Pediatric Intensive Care and Neuropediatrics, Comprehensive Center for Pediatrics, Medical University of Vienna, Währinger Gürtel 18-20, 1090 Vienna, Austria; Department of Pediatrics and Adolescent Medicine, Division of Neonatology, Pediatric Intensive Care and Neuropediatrics, Comprehensive Center for Pediatrics, Medical University of Vienna, Währinger Gürtel 18-20, 1090 Vienna, Austria; Department of Pediatrics and Adolescent Medicine, Division of Neonatology, Pediatric Intensive Care and Neuropediatrics, Comprehensive Center for Pediatrics, Medical University of Vienna, Währinger Gürtel 18-20, 1090 Vienna, Austria; Center for Health and Bioresources, Molecular Diagnostics, AIT Austrian Institute of Technology GmbH, Giefinggasse 4, 1210 Vienna, Austria; Center for Health and Bioresources, Molecular Diagnostics, AIT Austrian Institute of Technology GmbH, Giefinggasse 4, 1210 Vienna, Austria; Department of Pediatrics and Adolescent Medicine, Division of Neonatology, Pediatric Intensive Care and Neuropediatrics, Comprehensive Center for Pediatrics, Medical University of Vienna, Währinger Gürtel 18-20, 1090 Vienna, Austria; Department of Biomedical Imaging and Image-Guided Therapy, Division of Neuroradiology and Musculoskeletal Radiology, Medical University of Vienna, Währinger Gürtel 18-20, 1090 Vienna, Austria; Department of Biomedical Imaging and Image-Guided Therapy, Division of Neuroradiology and Musculoskeletal Radiology, Medical University of Vienna, Währinger Gürtel 18-20, 1090 Vienna, Austria; Department of Pediatrics and Adolescent Medicine, Division of Neonatology, Pediatric Intensive Care and Neuropediatrics, Comprehensive Center for Pediatrics, Medical University of Vienna, Währinger Gürtel 18-20, 1090 Vienna, Austria

**Keywords:** cerebral intraventricular hemorrhage, neonate, MRI scans, brainstem, software

## Abstract

**Objectives:**

This retrospective study aimed to identify quantitative magnetic resonance imaging markers in the brainstem of preterm neonates with intraventricular hemorrhages. It delves into the intricate associations between quantitative brainstem magnetic resonance imaging metrics and neurodevelopmental outcomes in preterm infants with intraventricular hemorrhage, aiming to elucidate potential relationships and their clinical implications.

**Materials and methods:**

Neuroimaging was performed on preterm neonates with intraventricular hemorrhage using a multi-dynamic multi-echo sequence to determine T1 relaxation time, T2 relaxation time, and proton density in specific brainstem regions. Neonatal outcome scores were collected using the Bayley Scales of Infant and Toddler Development. Statistical analysis aimed to explore potential correlations between magnetic resonance imaging metrics and neurodevelopmental outcomes.

**Results:**

Sixty preterm neonates (mean gestational age at birth 26.26 ± 2.69 wk; *n* = 24 [40%] females) were included. The T2 relaxation time of the midbrain exhibited significant positive correlations with cognitive (*r* = 0.538, *P* < 0.0001, Pearson’s correlation), motor (*r* = 0.530, *P* < 0.0001), and language (*r* = 0.449, *P* = 0.0008) composite scores at 1 yr of age.

**Conclusion:**

Quantitative magnetic resonance imaging can provide valuable insights into neurodevelopmental outcomes after intraventricular hemorrhage, potentially aiding in identifying at-risk neonates. Multi-dynamic multi-echo sequence sequences hold promise as an adjunct to conventional sequences, enhancing the sensitivity of neonatal magnetic resonance neuroimaging and supporting clinical decision-making for these vulnerable patients.

## Introduction

Intraventricular hemorrhage (IVH) is one of the most common neurologic complications in premature neonates and is considered the most frequent type of neonatal intracranial bleeding (Kenet et al. 2011; Deger et al. 2021). Approximately 15% to 20% of neonates born before 32 wk gestational age (GA; Szpecht et al. 2016) and 31% of infants born before 27 wk GA, respectively, are affected (Yeo et al. 2020). With 15 million premature infants born worldwide each year, IVH is a global health problem in this ever-increasing group of particularly fragile patients (Ream and Lehwald 2018; Walani 2020).

IVH usually originates in the ganglionic eminence—a highly vascularized zone, which is prone to bleeding due to hemodynamic imbalances in the preterm vascular system—and commonly enters the ventricular compartment (Langley et al. 2022). It can range from small amounts of intraventricular blood components to severe periventricular hemorrhage (Valdez Sandoval et al. 2019). The risk of severe hemorrhage and complications like posthemorrhagic ventricular dilatation (PHVD), and subsequent neurological impairment, increases with lower GA at delivery and lower birth-weight (Murphy et al. 2002; Navidi et al. 2022; Piccolo et al. 2022; Vohr 2022). Clinically, IVH may remain asymptomatic and can be detected by routine cranial ultrasound. Precise imaging diagnostics, a rapidly organized neonatal intensive care setting, and the earliest possible sufficient treatment of PHVD are crucial for optimal neurodevelopmental outcome in affected neonates (Ballabh and de Vries 2021).

Changes in brain myelination have been reported in neonates with IVH (van de Bor et al. 1989; Cheng and Ballabh 2022). The onset of IVH triggers axonal degeneration and compromises the integrity of oligodendrocyte progenitor cells, resulting in an altered myelination process within the white matter (Ballabh and de Vries 2021). This primarily impacts infratentorial structures, since myelinogenesis progresses along a cauda–cranial trajectory (Branson 2013). The myelination process in brainstem components reaches completion by the standard time of birth (Yakovlev 1967; Barkovich et al. 1988). In contrast to diagnostic approaches using sonography, magnetic resonance imaging (MRI) allows for the simultaneous assessment of the myelination status (Childs et al. 2001; Benders et al. 2014; Hinojosa-Rodriguez et al. 2017).

Quantitative MRI methods facilitate the assessment of cerebral growth and the maturation of the brain by leveraging specific relaxation characteristics and proton density (PD) as markers (Ferrie et al. 1999; Ding et al. 2004). The application of quantitative T1 and T2 mapping is advantageous in the qualitative evaluation of myelination in the neonatal brain (Deoni et al. 2011).

Synthetic Magnetic Resonance Imaging (SyMRI®) is a novel MR technique in which a single multi-dynamic multi-echo sequence (MDME) is used to generate a variety of imaging contrasts (Andica et al. 2019). It is effective for studying the neonatal brain (Schmidbauer, Geisl, et al. 2021b) and providing a more precise qualitative evaluation of myelination processes in preterm infants (Schmidbauer et al. 2019).

While the link between disrupted myelin development in neonates with IVH has been established, predictive models or biomarkers to estimate future outcome in preterm infants with a history of IVH are still lacking. In search of an easily applicable biomarker, we chose to assess SyMRI® of early myelinating structures in the neonatal brainstem for its predictive value for postnatal outcome.

In pursuit of our objective to explore the associations between brainstem MRI metrics and neurodevelopmental outcomes in preterm infants with IVH, we employed a retrospective observational approach, utilizing MDME sequences. The goal was to offer noninvasive indicators suitable for clinical application, toward a more precise and personalized diagnostic approach in these vulnerable patients.

## Materials and methods

This retrospective study was approved by the local Institutional Review Board. All study protocols and procedures were conducted in accordance with the Declaration of Helsinki (World Medical 2013). Written informed consent from the guardians was waived due to the retrospective character of the study.

This study builds upon previous research creating an MRI-based scoring system to predict outcome for patients with IVH (Goeral et al. 2022) as well as a study examining brain growth in neonates with IVH, including a cohort of 21 participants from the initial investigation (Steiner et al. 2023).

This study included neonates diagnosed with IVH who underwent MDME MRI sequences. Cases with major congenital anomalies (e.g. tetralogy of Fallot, omphalocele, gastroschisis, clubfeet, Duchenne muscular dystrophy, Hirschsprung’s disease, or Pierre Robin sequence) cerebral malformations (e.g. neural tube defects, Dandy–Walker malformation, Arnold–Chiari malformation, or holoprosencephaly), metabolic disorders (e.g. congenital hypothyroidism or phenylketonuria), chromosomal abnormalities, as well as death before follow-up, and cases with insufficient image quality, were excluded. Detailed clinical information on the neonates’ health, including any incidents of infections and comorbidities are listed in Supplementary Material Tables S1 to S3.

### Neonatal neuroimaging analysis

#### Image acquisition

Neuro MRIs were conducted following the established institutional feed-and-wrap protocol, as described in detail in (Buchmayer et al. 2022), on a Philips Ingenia 1.5 Tesla MR system (Philips Health Systems, Eindhoven, the Netherlands). The MRI was scheduled as exactly as possible on the day of the expected birth (median GA at MRI 37.50 wk [interquartile range {IQR} 36.33 to 38.97 wk]). To minimize potential distortions caused by motion, neonates were positioned on a vacuum mattress and received chloralhydrate via gastric tube. A standardized neonatal MR protocol (Supplementary Material Table S4) was used for the examinations.

MDME sequences (transversal plane; slice thickness: 4 mm; slice gap: 1 mm; field of view: 320 × 320; matrix 224 × 193; TE [echo time]: 12.5/100 ms; and TR [repetition time]: 3282.19) were performed using a 2-phase, repeated acquisition approach (Warntjes et al. 2008). In phase 1, a slice-selective saturation pulse with a flip angle of 120° was used to saturate a specific section. In phase 2, slice-selective refocusing pulses with flip angles of 180° and excitation pulses with flip angles of 90° generated a series of spin echoes for another section (Hagiwara et al. 2017; Kang et al. 2019).

The additional MDME sequences extended the standard protocol from 15:42 min by 5:54 min to a total of 21:06 min.

#### Image analysis

Diagnoses of IVH and grading (according to Papile; Papile et al. 1978) were made by an experienced neonatologist with 10 yr of experience (K.G.) on ultrasound and confirmed by an experienced neuroradiologist with 20 yr of experience with neonatal MRI (G.K.).

The MDME postprocessing software SyMRI® (Version 11.2.9, SyntheticMR AB, Linköping, Sweden) enabled the determination of T1 relaxation time (T1R), T2 relaxation time (T2R), and PD properties.

Two independent readers (M.S.Y. and P.K., both with at least 2 yr of experience in neonatal MRI), blinded for IVH grade and GA, placed regions of interest (ROIs) manually on SyMRI®-generated maps in the midbrain, pontine tegmentum, basis pontis, and the medulla oblongata (schematic illustration in Supplementary Material Fig. S1).

**Fig. 1:**
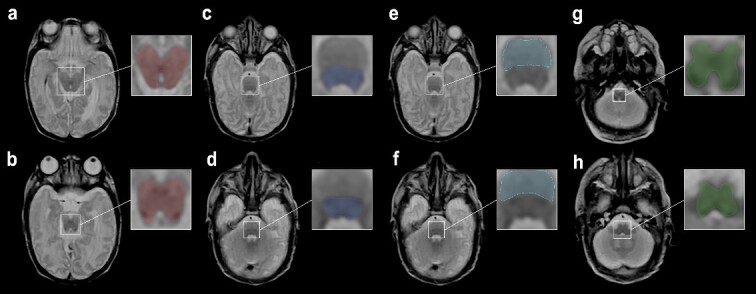
Female neonate with IVH grade III, born at 24 + 0 wk GA. MRI performed 93 days postpartum. ROIs were drawn on an MDME-based T2 contrast on a transversal plane. ROIs of the midbrain at the level of the a) superior colliculi b) inferior colliculi. c) Pontine tegmentum and e) basis pontis at the level of the superior olive. d) Pontine tegmentum and f) basis pontis at the level of the vestibular nuclei. Medulla oblongata at the level of the g) medial lemniscus (decussation) and h) inferior olivary nucleus.

For the midbrain, the slice at the level of the superior colliculi and the slice at the level of the inferior colliculi were identified on the transversal MDME sequence. An ROI was drawn for each, and intensity averages for T1R, T2R, and PD were calculated from the 2 ROIs. The same procedure was followed for the pontine tegmentum and basis pontis (level of the superior olive and level of the vestibular nuclei) and the medulla oblongata (level of the medial lemniscus and the inferior olivary nucleus, Fig. 1).

### Neurodevelopmental outcome

The German adaption of the Bayley Scales of Infant and Toddler Development 3^rd^ edition (Bayley-III) was assessed by a clinical psychologist with 30 yr of experience (R.F.) (Bayley 1969; Macha and Petermann 2015; Yi et al. 2018). The cognitive (CCS), language (LCS), and motor (MCS) composite scores were collected at 1 (median 367 [IQR 358 to 383] days) and 2 yr (median 737 [IQR 729 to 747] days) of term-equivalent age.

### Statistics

Statistical analysis was performed using SPSS for MacOS (Version 29, IBM, New York, United States of America).

Pearson’s correlation analyses were conducted to detect associations between the quantitative MRI measures, assessed at term-equivalent age, and outcome scores (cognitive, language, and MCS) collected at 1 and 2 yr of corrected age.

One-way ANOVA and partial eta squares were calculated for the correlation between T1R, T2R, and PD and the development of PHVD, as well as the need for neurosurgical intervention (Adams and Conway 2014).

Corrections for multiple testing were waived due to the small sample size and the increased risk of a type II error (Rothman 1990).

Interrater reliability between the 2 raters was calculated using the intraclass correlation coefficient (ICC; average measures, 2-way mixed model, absolute agreement, Supplementary Material Table S5). According to Koo and Li, an ICC between 0.75 and 0.9 was considered good, and >0.9 excellent agreement (Koo and Li 2016). Demonstrated results are based on measures performed by rater 1 (M.S.Y.).

A *P*-value equal to or below 0.05 was considered to indicate significant results.

## Results

### Patient cohort

**Fig. 2:**
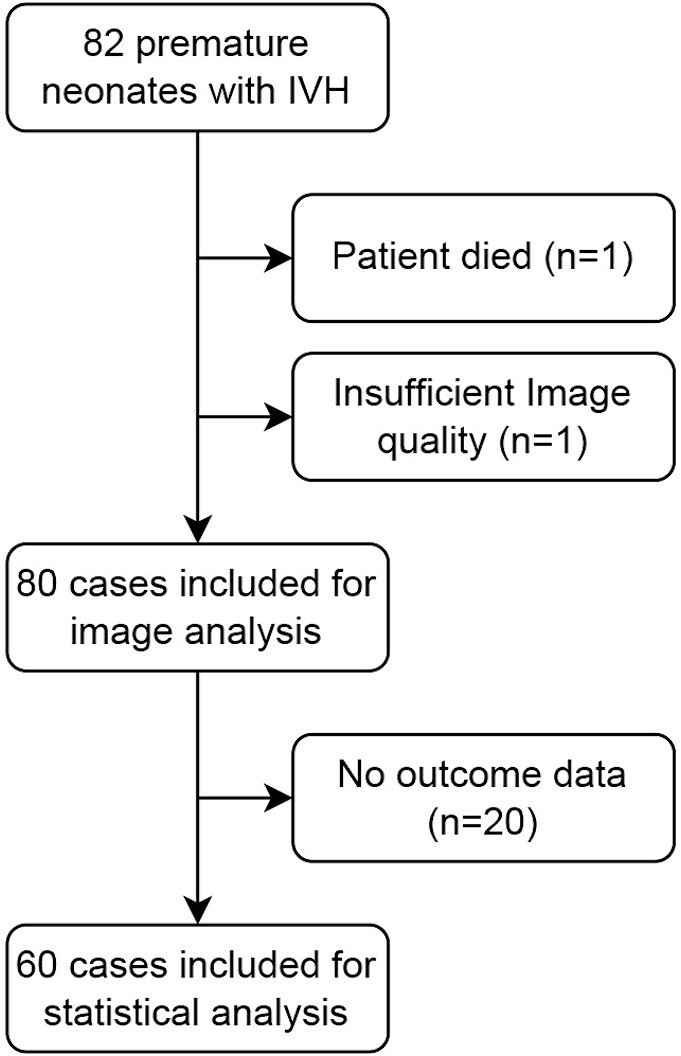
Flowchart with patient cohort and excluded cases.

Neuroimaging was performed at term-equivalent age in 82 neonates with IVH born between October 2017 and August 2022. After a conscientious review of imaging data and neonatal outcome parameters, 60 subjects were finally included in this study (Fig. 2). All subjects were born between 22 + 6 and 31 + 6 wk GA. Twenty-four (40%) of the subjects were female. All neonates were born prematurely between 22 + 6 and 31 + 6 wk GA (mean GA at birth 26.26 ± 2.69 wk standard deviation). Fifty-one (85%) infants were born extremely preterm according to World Health Organization criteria (<28 wk GA; Cheong et al. 2017), and 9 (15%) were very preterm (28 to < 32 wk).

According to Papile (Papile et al. 1978), IVH grade I was diagnosed in *n* = 10 (16.7%); IVH grade II in *n* = 21 (35%); IVH grade III in *n* = 15 (25%); and IVH grade IV (periventricular hemorrhagic infarction (PVHI)) in *n* = 14 (23.3%). Twenty cases (33.3%) developed PHVD (3× IVH I, 9× IVH II, 4× IVH III, and 4× IVH IV/PVHI) and 13 cases (21.7%) required neurosurgical intervention (3× IVH I, 5× IVH II, 2× IVH III, 3× IVH IV/PVHI).

### Correlation between neonatal neuroimaging and neurodevelopmental outcome

Outcome parameters collected at 1 yr corrected age were available in *n* = 53/60 (88.3%), at 2 yr corrected age in *n* = 46/60 (76.7%), and collected at 1 *and* 2 yr corrected age in *n* = 39/60 (65.0%) in the final patient cohort analyzed.

#### Midbrain

T2R in the midbrain demonstrated a significant positive correlation with CCS after 1 yr corrected age (*r* = 0.538, *P* < 0.0001) and 2 yr (*r* = 0.380, *P* = 0.009), LCS after 1 yr (*r* = 0.449, *P* = 0.0008) and 2 yr (*r* = 0.307, *P* = 0.038), and MCS after 1 yr (*r* = 0.530, *P* < 0.0001) and 2 yr (*r* = 0.433, *P* = 0.003).

The midbrain showed a (highly) significant correlation between PD measurements and CCS after 1 yr (*r* = 0.305, *P* = 0.028) and 2 yr (*r* = 0.293, *P* = 0.048), LCS after 1 yr (*r* = 0.434, *P* = 0.001), and MCS after 1 yr (*r* = 0.300, *P* = 0.031).

#### Basis pontis

Similarly, T1R in the basis pontis was significantly correlated with CCS after 1 yr (*r* = 0.433, *P* = 0.002) and 2 yr (*r* = 0.359, *P* = 0.018), LCS after 1 yr (*r* = 0.432, *P* = 0.002), and MCS after 1 yr (*r* = 0.466, *P* = 0.0007) and 2 yr (*r* = 0.327, *P* = 0.032).

The basis pontis showed significant correlations between T2R measurements and CCS after 1 yr (*r* = 0.298, *P* = 0.037) and MCS after 1 yr (*r* = 0.349, *P* = 0.014).

Significant correlations were also found between PD measurements and CCS after 1 yr (*r* = 0.294, *P* = 0.04) and after 2 yr CCS (*r* = 0.333, *P* = 0.029).

#### Medulla oblongata and pontine tegmentum

There was no significant correlation between the measured metrics in the medulla oblongata or the pontine tegmentum and neurodevelopmental outcome (for a detailed overview of the correlated values, see Table 1).

**Table 1:** Correletations between neurodevelopmental outcome after 1 yr of life (CCS 1, LCS 1, and MCS 1), 2 yr of life (CCS 2, LCS 2, and MCS 2), and metric relaxation parameters based on MDME sequences.

		CCS 1	LCS 1	MCS 1	CCS 2	LCS 2	MCS 2
Medulla oblongata T1R	Pearson correlation	0.209	0.139	0.271	0.105	0.026	0.157
	Sig. (2-tailed)	0.137	0.327	0.052	0.489	0.866	0.297
Medulla oblongata T2R	Pearson correlation	0.191	0.126	0.190	0.036	−0.008	0.008
	Sig. (2-tailed)	0.175	0.375	0.178	0.810	0.958	0.956
Medulla oblongata PD	Pearson correlation	0.067	0.075	0.022	−0.062	0.026	−0.034
	Sig. (2-tailed)	0.636	0.598	0.877	0.683	0.866	0.823
Pontine tegmentum T1R	Pearson correlation	0.149	0.113	0.162	−0.012	−0.112	0.018
	Sig. (2-tailed)	0.293	0.425	0.252	0.935	0.459	0.905
Pontine tegmentum T2R	Pearson correlation	0.199	0.111	0.258	0.039	−0.034	0.048
	Sig. (2-tailed)	0.157	0.434	0.064	0.795	0.820	0.752
Pontine tegmentum PD	Pearson correlation	0.181	0.162	0.083	0.057	0.076	−0.024
	Sig. (2-tailed)	0.198	0.252	0.561	0.704	0.617	0.876
Basis pontis T1R	Pearson correlation	**0.433** ^**a**^	**0.432** ^**a**^	**0.466** ^**a**^	**0.359** ^**b**^	0.189	**0.327** ^**b**^
	Sig. (2-tailed)	**0.002**	**0.002**	**0.001**	**0.018**	0.225	**0.032**
Basis pontis T2R	Pearson correlation	**0.298** ^**b**^	0.235	**0.349** ^**b**^	0.166	0.155	0.091
	Sig. (2-tailed)	**0.037**	0.104	**0.014**	0.287	0.321	0.563
Basis pontis PD	Pearson correlation	**0.294** ^**b**^	0.280	0.188	**0.333** ^**b**^	0.253	0.230
	Sig. (2-tailed)	**0.040**	0.052	0.197	**0.029**	0.101	0.137
Midbrain T1R	Pearson correlation	0.201	0.184	0.162	0.068	−0.061	0.126
	Sig. (2-tailed)	0.152	0.192	0.252	0.652	0.685	0.406
Midbrain T2R	Pearson correlation	**0.538** ^**a**^	**0.449** ^**a**^	**0.530** ^**a**^	**0.380** ^**a**^	**0.307** ^**b**^	**0.433** ^**a**^
	Sig. (2-tailed)	**0.000**	**0.001**	**0.000**	**0.009**	**0.038**	**0.003**
Midbrain PD	Pearson correlation	**0.305** ^**b**^	**0.434** ^**a**^	**0.300** ^**b**^	**0.293** ^**b**^	0.225	0.268
	Sig. (2-tailed)	**0.028**	**0.001**	**0.031**	**0.048**	0.133	0.072

Significant results are shown in bold.

^a^Correlation is significant at the 0.01 level (2-tailed).

^b^Correlation is significant at the 0.05 level (2-tailed).

### Correlation between the severity of hemorrhage and neurodevelopmental outcome

Subdivided by severity of hemorrhage according to Papile, the low-grade group (IVH I and II) showed significant correlations only between the T2R determined in the midbrain and the CCS collected at 1 yr corrected age (*r* = 0.481, *P* = 0.013; Supplementary Material Table S6).

The high-grade group (IVH III and IV/PVHI) showed significance in the correlation between T2R of the midbrain and CCS after 1 yr (*r* = 0.506, *P* = 0.008), LCS after 1 yr (*r* = 0.444, *P* = 0.023), and MCS after 1 yr (*r* = 0.624, *P* = 0.001) and 2 yr (*r* = 0.473, *P* = 0.0023). In this group, there was a significant correlation between PD of the midbrain and LCS after 1 yr (*r* = 0.431, *P* = 0.028).

T1R of the basis pontis showed highly significant correlations with CCS after 1 yr (*r* = 0.507, *P* = 0.010), LCS after 1 yr (*r* = 0.581, *P* = 0.002), and MCS after 1 yr (*r* = 0.592, *P* = 0.002; Supplementary Material Table S7).

### Correlation between neonatal neuroimaging and development of PHVD

Significant correlations were shown between the measured quantitative metrics of the medulla oblongata, pontine tegmentum, basis pontis, and midbrain, the subsequent development of PHVD, and the need for neurosurgical intervention (Table 2). These correlations were most pronounced for the T2R metrics (medulla oblongata T2R-neurosurgery: *P =* 0.0002, *η^2^* = 0.167; pontine tegmentum T2R-neurosurgery: *P <* 0.0001, *η^2^* = 0.200; midbrain T2R-PHVD: *P <* 0.0001, *η^2^* = 0.197; midbrain T2R-neurosurgery: *P <* 0.0001, *η^2^* = 0.188).

**Table 2:** Correlation between relaxation parameters and potential occurrence of PHVD or need for neurosurgical intervention.

		Medulla oblongata T1R	Medulla oblongata T2R	Medulla oblongata PD	Pontine tegmentum T1R	Pontine tegmentum T2R	Pontine tegmentum PD	Basis pontis T1R	Basis pontis T2R	Basis pontis PD	Midbrain T1R	Midbrain T2R	Midbrain PD
PHVD	Partial eta squared	**0.104**	**0.077**	0.003	**0.101**	**0.122**	0.010	**0.140**	0.048	0.002	**0.059**	**0.197**	**0.114**
	Sig. (2-tailed)	**0.004**	**0.014**	0.624	**0.005**	**0.002**	0.395	**0.005**	0.107	0.764	**0.032**	**<0.0001**	**0.002**
Neurosurgery	Partial eta squared	**0.070**	**0.167**	0.006	**0.090**	**0.200**	0.020	0.055	**0.134**	0.000	**0.057**	**0.188**	**0.176**
	Sig. (2-tailed)	**0.019**	**0.0002**	0.483	**0.008**	**<0.0001**	0.212	0.086	**0.006**	0.984	**0.035**	**<0.0001**	**0.0001**

Significant results are shown in bold.

### Intraclass correlation analysis

The ICC showed almost excellent agreement between both raters for all structures and metrics ranging from 0.844 (95% CI 0.711 to 0.910) to 0.991 (95% CI 0.985 to 0.995; detailed analysis in Supplementary Material Table S5).

## Discussion

Our study embarked on addressing the gap in predicting neurodevelopmental outcome in preterm infants with IVHs, a prevalent concern that poses significant challenges for early intervention and therapeutic strategies.

Easily applicable, reliable clinical-radiologic imaging biomarkers in the prognostic assessment of outcome after IVH are relevant for further treatment stratification, therapeutic support, and parental counseling.

A recent study successfully explored the utility of quantitative MRI metrics in predicting neurodevelopmental outcomes in extremely preterm neonates (Schmidbauer et al. 2024).

Here, we focused on early myelinating structures in the neonatal brainstem, which harbors primary corticospinal connections as well as primary neurophysiologic functions, using a rather novel quantitative MRI approach and its relation to future outcome in a comparably large group of preterm neonates with IVH.

Positive correlations between outcomes with T2R and PD values were particularly pronounced in the midbrain. According to the literature, increased relaxometric properties and PD values are considered to indicate lower myelination states (Lee et al. 2018; Schmidbauer et al. 2019). However, the results observed in this study suggest that MRI metrics associated with more advanced states of myelination at term-equivalent age correlate with generally poorer future outcomes in terms of cognitive, language, and motor performance (schematic overview in Table 3).

**Table 3:** A simplified overview of the quantified T1R, T2R, and PD values and their significance in the studied patient population.

	T1R	T2R	PD
Better neurodevelopmental outcome	▲	▲	▲
Worse neurodevelopmental outcome	▼	▼	▼
Accelerated myelination	▼	▼	▼

Higher T1R, T2R, and PD metrics are based on an accelerated myelination of the investigated regions of the brainstem. This accelerated myelination is associated with poorer neurodevelopmental outcome. Conversely, neonates with lower T1R, T2R, and PD values showed better neurodevelopmental outcome.

Our observations and those of others support the hypothesis that achieving a more mature state of the brainstem and white matter development earlier is associated with poorer neurodevelopmental outcome. This has been explained by studies showing accelerated brain development in preterm neonates in specific neurostructural regions, associated with excessive connectivity and myelination (Alexander et al. 2019; Pittet et al. 2019; Zun et al. 2021). Kim et al. showed that more mature states of myelination of the preterm brain appear to lead to an impaired cognitive outcome (Kim et al. 2022).

As myelination follows a path from caudally to rostrally (Branson 2013), the present study focused on quantitative metrics of the brainstem, as this structure holds relatively large amounts of myelin from 20 gestational wk onward (Tanaka et al. 1995).

Throughout brain maturation, there is evidence suggesting that relaxation times undergo dynamic changes (Lee et al. 2018; Schmidbauer et al. 2022). Notably, the process of myelination seems to play a crucial role in altering T1R/T2R and PD during the fetal period and early infancy (Dubois et al. 2014; Wang et al. 2019).

It is known that T1R already experiences a decline during the pre-myelination stage, which refers to the initial interactions between H_2_O and myelin building blocks (such as glycolipids and cholesterol) at the onset of myelinogenesis. However, T2R does not exhibit considerable shortening until fully developed myelin sheaths are formed (Barkovich et al. 1992; Wang et al. 2019). Therefore, lower relaxation time metrics are indicative of more advanced stages of myelination (Lee et al. 2018; Schmidbauer, Dovjak, et al. 2021a).

Consequently, our quantification focused on regions in the brainstem where significant advancements in brain myelination are known to occur during early developmental stages.

The midbrain, which revealed the strongest correlations between quantitative MR measures and long-term outcomes, holds certain structures that appear to play an important role with respect to future neurodevelopment. The present study indicates that the maturation of this region, as observed through quantitative MRI, is strongly associated with neurodevelopmental outcome. Various studies have demonstrated that the midbrain undergoes significant morphological changes in response to ongoing brain maturation, as evidenced by signal intensity changes on MRI. These changes are most likely attributed to ongoing myelination processes and ferric iron deposition. Our findings, however, further indicate that the myelination state of this region is strongly associated with neurodevelopmental outcome (Martin et al. 1991; Counsell et al. 2002).

Several structures in the midbrain undergo early myelination: the inferior colliculi are first detectable on T2-weighted fast spin-echo MR images in preterm neonates as early as the time-equivalent GA 25 onward, the medial longitudinal fasciculi as early as time-equivalent GA 29, and the medial lemnisci as early as time-equivalent GA 30 (Counsell et al. 2002). This region houses nuclei and fiber tracts crucial for the development of linguistic, cognitive, and motor skills, highlighting their significant impact on sensorimotor feedback loops (Duerden et al. 2015; Bissonette and Roesch 2016; Gut and Mena-Segovia 2019; Basso et al. 2021).

Moreover, the inferior colliculi house a crucial functional site that governs the auditory pathway. Disruptions in this area can lead to delayed language acquisition, reduced cognitive abilities, and impaired socio-developmental advancement (Ream and Lehwald 2018).

Furthermore, MR measurements of the basis pontis correlated positively with neurodevelopmental outcomes in our study. Highly significant positive correlations were found between the relaxation parameters measured on T1R in the basis pontis and cognitive, language, as well as motor outcomes. This may be attributed to the process of myelination of the tegmentum pontis being almost finished at term, which does not apply to the basis pontis (Brody et al. 1987; Tate et al. 2015). The sharp postnatal increase in oligodendrocyte generation and proliferation of Olig2+ cells is closely associated with the rapid progress of motor skills observed during the early months of life (Brody et al. 1987; Tate et al. 2015). Simultaneously, the development and volumetric enlargement of the corticospinal tract, passing through the base of the pons, occur in conjunction with the aforementioned processes. The premature maturation of these brain structures appears to have a detrimental effect on the flexibility and plasticity of the pyramid pathway, thereby negatively affecting neurodevelopment.

In our study, neonates with low-grade IVH (grades I and II according to Papile) showed a single significant positive correlation between reduced midbrain T2R values and impaired cognitive outcome at 1 yr. By the age of 2, however, these infants had displayed comparable neurodevelopment, pointing toward effective compensatory mechanisms during further development. This underlines that while MDME-based metrics can sensitively predict early developmental challenges, these challenges appear to represent a transient developmental delay rather than lasting adverse outcomes.

For grade III and IV/PVHI hemorrhages, however, MDME-based markers indicate associations with long-term prognosis (in terms of cognitive, language, and motor development) as early as 1 yr of corrected age.

In the past, it was generally believed that IVH grades I and II, which can be difficult to detect on ultrasound (Buchmayer et al. 2022), were associated with favorable outcome, while grades III and IV/PVHI were correlated with adverse results. Our study, however, indicates that a more nuanced distinction is possible with the aid of quantitative MRI, even within the high-grade IVH categories. Novel scoring systems are emerging to assist clinicians in achieving a more refined prognosis (Goeral et al. 2022). Furthermore, our study has illustrated the association between metric correlations in neonatal brainstems and neurodevelopmental outcomes.

The highly significant correlations between relaxation parameters and the later occurrence of PHVD or the need for neurosurgical intervention, respectively, were again most pronounced in the midbrain (Table 2). While brainstem metric measures at term-equivalent age are not yet viable as a clinical decision criterion for neurosurgical procedures, their significant correlations make them valuable candidates for inclusion in future models. These models aim to enhance clinical decision-making and improve outcome predictions.

Clinicians could consider these metrics to identify infants at higher risk of adverse outcome early, enabling the initiation of targeted early intervention programs. Infants exhibiting patterns of accelerated myelination might benefit from closer neurodevelopmental monitoring and early engagement in therapies designed to enhance cognitive, language, and motor skills (Ballabh and de Vries 2021; Sant et al. 2021).

The degree of brainstem involvement indicated by MRI could guide the intensity and nature of therapeutic interventions, ranging from physical therapy to more specialized neurodevelopmental therapies, e.g. cognitive developmental therapy, occupational therapy, speech and language therapy, or neurodevelopmental treatment.

MDME sequences offer the advantage of providing multiple contrasts while providing quantitative maps based on a single scan. Thus, MDME sequences may become an important component for routine MRIs for premature neonates in the future to shorten the overall examination time in this vulnerable patient population.

The MDME sequences displayed substantial interrater reliability in our study and their modality reliability, as reported in the existing literature, aligns with that of conventional sequences (Schmidbauer, Geisl, et al. 2021b). This observation is particularly relevant for neonates, given that their MRI scans frequently suffer from motion artifacts.

These findings underscore the promising capacity of MDME sequences to complement conventional MRI sequences in neonatal imaging.

### Strengths and limitations

The excellent and consistent intraclass coefficients between the 2 raters confirm that SyMRI® is a reliable and accurate tool for the quantification of MR metrics in the neonatal brainstem. The presented analysis may be performed rapidly, allowing incorporation into the clinical routine. Future studies could explore the fully automated analysis and interpretation of the examined structures.

However, our study had limitations that should be considered. First, the study focused solely on a restricted number of early-myelinating areas in the brainstem, neglecting the exploration of associations between quantitative metrics in pre-myelinating regions and neurodevelopmental outcomes.

Furthermore, this study did not explore potential correlations between neurodevelopmental outcome and extracranial effects of preterm birth.

In this study, the use of the Bayley Scales of Infant and Toddler Development presents limitations that need consideration. Primarily, the Bayley scales offer quantitative outcomes that may not fully reflect the qualitative aspects of neurodevelopment, particularly in motor skills. Children may achieve acceptable scores yet still exhibit notable developmental challenges, especially in motor function (Burakevych et al. 2017).

Due to the novelty of this technique, MDME sequences are still rarely performed in the daily clinical setting. As a result, neonatal norm values from a (larger) patient population are not yet available. This study further investigated the relationship between T1R/T2R metrics and neurodevelopmental outcome in neonates with IVH but did not include a healthy control population.

Furthermore, outcome parameters were not available for all patients at 2 yr; therefore, the results are based primarily on 1 yr outcome.

## Conclusion

Quantitative MRI of the brainstem included in routine diagnostics of preterm infants, offers valuable prognostic information, facilitating the early identification of neonates at risk for complications and unfavorable long-term outcome following IVH.

MDME sequences show the potential to complement or replace conventional sequences in the future, and thus, refine the current gold standard in neonatal MR neuroimaging.

## Supplementary Material

Supplementary_Material_bhae189
