# Supplementary material for: Neurodevelopmental outcome in preterm infants with intraventricular hemorrhages: the potential of quantitative brainstem MRI
Source: Cereb Cortex. 2024 May 7;34(5):bhae189. doi: 10.1093/cercor/bhae189 (PMC11077078; doi:10.1093/cercor/bhae189)
Supplement: Supplementary_Material_bhae189 [file supplementary_material_bhae189.docx]

**Supplementary Material**

**Figures**


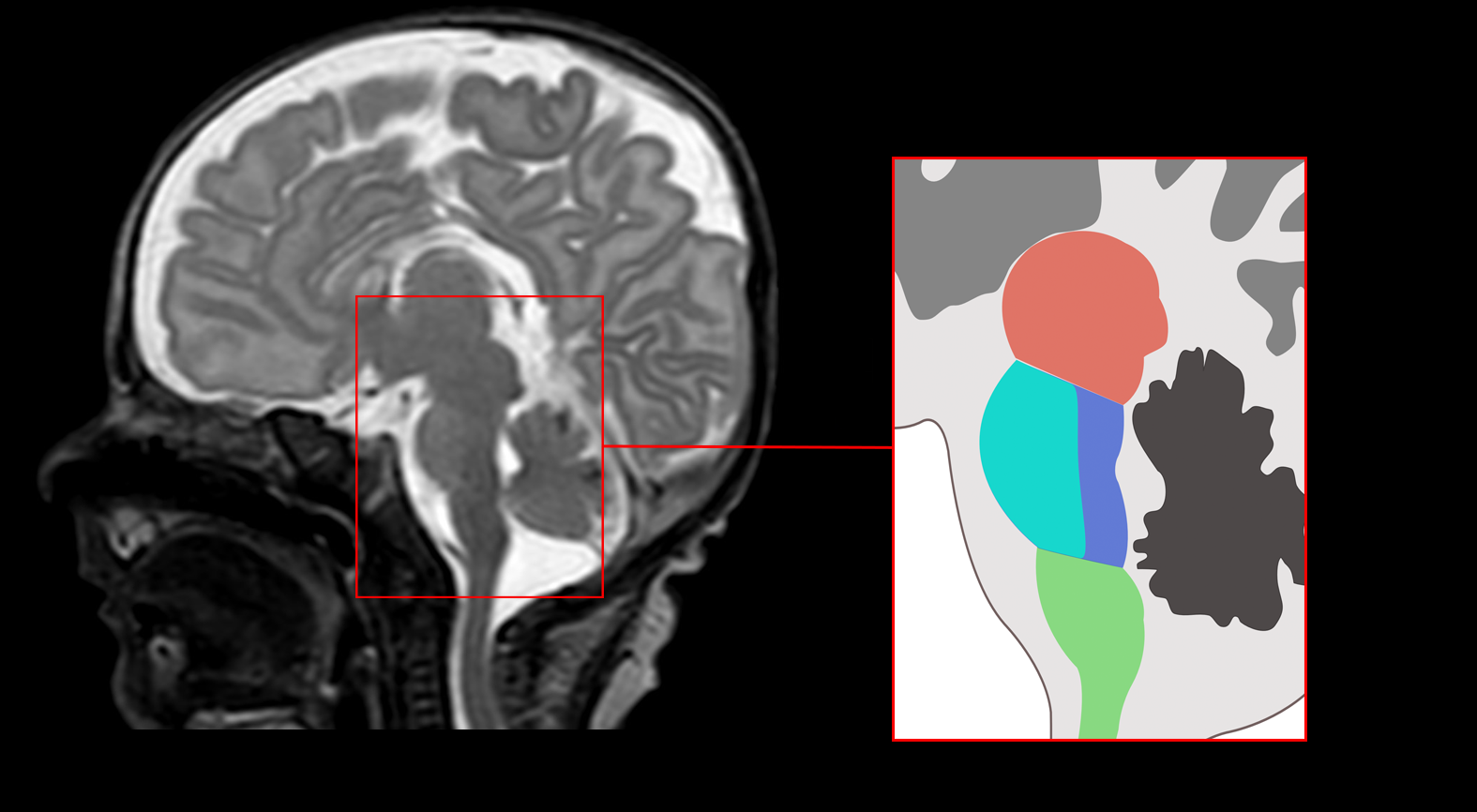


**Figure 1:** Schematic representation of the measured structures in the brainstem (T2-weighted sagittal plane). From top to bottom: Midbrain (red), base of the pons (turquoise), tegmentum of the pons (blue), medulla oblongata (green).

**Tables**

| Condition | Number of Cases (Percentage) |
| --- | --- |
| Perinatal Asphyxia | 7 (12.1%) |
| Seizures | 13 (21.7%) |
| Meningitis, Ventriculitis, Encephalitis | 2 (3.3%) |
| Periventricular Leukomalacia | 5 (8.3%) |

**Table 1:** Additional Brain Pathologies and Neurologic Problems During Neonatal Course

| Morbidity | Number of Cases (Percentage) |
| --- | --- |
| Bronchopulmonary Dysplasia (BPD) | 17 (28.3%) |
| Retinopathy of Prematurity (ROP) Grade ≥2 | 6 (10.0%) |
| Persistent Ductus Arteriosus (PDA) | 4 (6.7%) |
| Necrotising Enterocolitis Requiring Surgery | 2 (3.3%) |
| Intestinal Perforation (not associated with NEC) | 3 (5.0%) |
| Other Abdominal Problems Requiring Surgery | 5 (8.3%) |

**Table 2:** Additional Neonatal Morbidities

| Parameter | Number of Cases (Percentage) |
| --- | --- |
| Blood Culture Positive Sepsis (Any) | 18 (30.0%) |
| Early-Onset Sepsis | 4 (7.1%) |
| Late-Onset Sepsis | 15 (25.0%) |
| Marker | **Mean (Range)** |
| CRP Day 1 of Life | 0.30 (0.03-0.30) mg/dL |
| IL-6 Day 1 of Life | 50.0 (21.0-223.1) pg/mL |
| CRP Day 2-7 of Life | 0.60 (0.23-1.10) mg/dL |
| IL-6 Day 2-7 of Life | 31.0 (15.4-76.6) pg/mL |

**Table 3:** Microbiology and Infection Parameters. The pathogens identified in early-onset sepsis were 2x Escherichia coli, 1x Klebsiella pneumoniae, and 1x Candida albicans; in late-onset sepsis 10x various Staphylococci (partly in combination with other pathogens), 3x Escherichia coli, 1x Streptococci, and 1x Klebsiella pneumoniae).

| Sequence | Plane | TR (ms) | TE (ms) | Voxel Size (mm) | FOV (mm) | Matrix (slices) | AT (min) |
| --- | --- | --- | --- | --- | --- | --- | --- |
| T1 3D | Sagittal | 25 | 7.6 | 0.75x0.75x2.00 | 120x120x99 | 160x160x99 | 3:46 |
| T1 SE | Transversal | 400 | 15 | 0.83x1.05x3.00 | 120x120x90 | 144x115x30 | 3:07 |
| T2 TSE | Transversal | 3000 | 140 | 0.94x1.06x3.00 | 120x120x102 | 128x113x34 | 1:48 |
| T2 TSE | Coronal | 3000 | 140 | 0.94x1.06x3.00 | 110x110x108 | 116x103x36 | 1:48 |
| T2 TSE | Sagittal | 3000 | 140 | 0.94x1.06x3.00 | 120x120x108 | 128x113x36 | 1:48 |
| SWI | Transversal | 51 | 12 | 0.85x1.00x2.00 | 170x139x90 | 200x138x90 | 3:35 |
| MDME | Transversal | 3309 | 13 | 0.9x1x4 | 200x165x109 | 224x159x22 | 5:24 |

**Table 4**: Standardized neonatal MR protocol with additional MDME sequence.
*AT* = acquisition time, *DWI* = diffusion-weighted imaging, *MDME* = multi-dynamic multi-echo sequence, *mm* = millimiters, *ms* = milli seconds, *SE* = spin echo, *SWI* = susceptibilits-weighted imaging, *TE* = echo time, *TR* = reception time, *TSE* = turbo spin echo

|  |  | 95% CI | |
| --- | --- | --- | --- |
|  | Interclass Correlation Coefficient | Lower Bound | Upper Bound |
| Medulla oblongata T1R | .988 | .973 | .994 |
| Medulla oblongata T2R | .977 | .963 | .985 |
| Medulla oblongata PD | .862 | .758 | .918 |
| Pontine Tegmentum T1R | .901 | .840 | .937 |
| Pontine Tegmentum T2R | .937 | .893 | .962 |
| Pontine Tegmentum PD | .844 | .711 | .910 |
| Basis Pontis T1R | .991 | .985 | .995 |
| Basis Pontis T2R | .938 | .892 | .964 |
| Basis Pontis PD | .970 | .949 | .983 |
| Midbrain T1R | .986 | .979 | .991 |
| Midbrain T2R | .948 | .919 | .967 |
| Midbrain PD | .943 | .910 | .964 |

**Table 5:** Interclass Correlation for both readers (Average measures, Two-Way Mixed, Absolute Agreement).
*PD* = Proton density, *T1R* = T1 relaxation time, *T2R* = T2 relaxation time

|  | | CCS 1 | LCS 1 | MCS 1 | CCS 2 | LCS 2 | MCS 2 |
| --- | --- | --- | --- | --- | --- | --- | --- |
| Medulla Oblongata T1R | Pearson Correlation | 0.064 | -0.115 | 0.058 | 0.156 | 0.199 | 0.065 |
|  | Sig. (2-tailed) | 0.756 | 0.576 | 0.778 | 0.477 | 0.361 | 0.767 |
| Medulla Oblongata T2R | Pearson Correlation | 0.151 | 0.085 | -0.011 | -0.097 | 0.027 | -0.156 |
|  | Sig. (2-tailed) | 0.460 | 0.681 | 0.959 | 0.658 | 0.903 | 0.477 |
| Medulla Oblongata PD | Pearson Correlation | -0.087 | 0.049 | -0.143 | 0.013 | 0.118 | -0.100 |
|  | Sig. (2-tailed) | 0.673 | 0.813 | 0.486 | 0.954 | 0.592 | 0.649 |
| Pontine Tegmentum T1R | Pearson Correlation | 0.006 | -0.115 | -0.037 | -0.037 | -0.161 | -0.082 |
|  | Sig. (2-tailed) | 0.978 | 0.576 | 0.856 | 0.867 | 0.464 | 0.711 |
| Pontine Tegmentum T2R | Pearson Correlation | 0.162 | -0.150 | 0.075 | 0.120 | 0.124 | 0.055 |
|  | Sig. (2-tailed) | 0.430 | 0.464 | 0.716 | 0.586 | 0.573 | 0.804 |
| Pontine Tegmentum PD | Pearson Correlation | 0.152 | 0.250 | -0.112 | 0.365 | 0.413 | 0.177 |
|  | Sig. (2-tailed) | 0.460 | 0.219 | 0.585 | 0.087 | 0.050 | 0.420 |
| Basis Pontis T1R | Pearson Correlation | 0.237 | 0.065 | 0.189 | 0.260 | 0.026 | 0.137 |
|  | Sig. (2-tailed) | 0.265 | 0.763 | 0.376 | 0.254 | 0.911 | 0.555 |
| Basis Pontis T2R | Pearson Correlation | 0.309 | 0.129 | 0.316 | 0.408 | 0.395 | 0.273 |
|  | Sig. (2-tailed) | 0.141 | 0.547 | 0.133 | 0.067 | 0.076 | 0.232 |
| Basis Pontis PD | Pearson Correlation | 0.239 | 0.245 | 0.037 | 0.302 | 0.243 | 0.249 |
|  | Sig. (2-tailed) | 0.260 | 0.249 | 0.862 | 0.183 | 0.289 | 0.276 |
| Midbrain T1R | Pearson Correlation | 0.128 | 0.112 | -0.006 | -0.085 | -0.226 | -0.061 |
|  | Sig. (2-tailed) | 0.533 | 0.585 | 0.976 | 0.698 | 0.301 | 0.781 |
| Midbrain T2R | Pearson Correlation | **.481*** | 0.247 | 0.176 | 0.272 | 0.312 | 0.172 |
|  | Sig. (2-tailed) | **0.013** | 0.224 | 0.390 | 0.209 | 0.148 | 0.431 |
| Midbrain PD | Pearson Correlation | 0.067 | 0.276 | -0.001 | 0.121 | 0.037 | 0.073 |
|  | Sig. (2-tailed) | 0.746 | 0.173 | 0.995 | 0.584 | 0.868 | 0.742 |
| **. Correlation is significant at the 0.01 level (2-tailed). | | | | | | | |
| *. Correlation is significant at the 0.05 level (2-tailed). | | | | | | | |

**Table 6:** Correlation between measured imaging parameters and clinical outcome parameters for the group of low-grade IVH (IVH I & II).

*CCS* 1/2 = Cognitive composite score after 1/2 years of life, *LCS* = Language composite score after 1/2 years of life, *MCS* = Motor composite score after 1/2 years of life

|  | | CCS 1 | LCS 1 | MCS 1 | CCS 2 | LCS 2 | MCS 2 |
| --- | --- | --- | --- | --- | --- | --- | --- |
| Medulla Oblongata T1R | Pearson Correlation | 0.270 | 0.209 | 0.354 | 0.127 | -0.052 | 0.253 |
|  | Sig. (2-tailed) | 0.183 | 0.306 | 0.076 | 0.563 | 0.814 | 0.243 |
| Medulla Oblongata T2R | Pearson Correlation | 0.130 | 0.058 | 0.223 | 0.047 | -0.082 | 0.002 |
|  | Sig. (2-tailed) | 0.527 | 0.778 | 0.273 | 0.831 | 0.709 | 0.993 |
| Medulla Oblongata PD | Pearson Correlation | 0.267 | 0.100 | 0.148 | -0.033 | -0.015 | 0.161 |
|  | Sig. (2-tailed) | 0.188 | 0.627 | 0.472 | 0.880 | 0.945 | 0.464 |
| Pontine Tegmentum T1R | Pearson Correlation | 0.126 | 0.147 | 0.261 | -0.122 | -0.130 | -0.014 |
|  | Sig. (2-tailed) | 0.541 | 0.474 | 0.199 | 0.581 | 0.555 | 0.949 |
| Pontine Tegmentum T2R | Pearson Correlation | 0.137 | 0.129 | 0.279 | -0.081 | -0.183 | -0.022 |
|  | Sig. (2-tailed) | 0.505 | 0.531 | 0.167 | 0.712 | 0.403 | 0.921 |
| Pontine Tegmentum PD | Pearson Correlation | 0.111 | 0.027 | 0.141 | -0.290 | -0.333 | -0.169 |
|  | Sig. (2-tailed) | 0.590 | 0.895 | 0.492 | 0.179 | 0.120 | 0.442 |
| Basis Pontis T1R | Pearson Correlation | **.507**** | **.581**** | **.592**** | 0.372 | 0.301 | 0.385 |
|  | Sig. (2-tailed) | **0.010** | **0.002** | **0.002** | 0.089 | 0.173 | 0.077 |
| Basis Pontis T2R | Pearson Correlation | 0.280 | 0.251 | 0.346 | 0.013 | 0.019 | -0.029 |
|  | Sig. (2-tailed) | 0.175 | 0.227 | 0.090 | 0.956 | 0.933 | 0.897 |
| Basis Pontis PD | Pearson Correlation | 0.335 | 0.309 | 0.298 | 0.421 | 0.279 | 0.283 |
|  | Sig. (2-tailed) | 0.101 | 0.132 | 0.148 | 0.051 | 0.208 | 0.202 |
| Midbrain T1R | Pearson Correlation | 0.173 | 0.176 | 0.318 | 0.107 | 0.101 | 0.177 |
|  | Sig. (2-tailed) | 0.397 | 0.391 | 0.113 | 0.626 | 0.647 | 0.419 |
| Midbrain T2R | Pearson Correlation | **.506**** | **.444*** | **.624**** | 0.356 | 0.243 | **.473*** |
|  | Sig. (2-tailed) | **0.008** | **0.023** | **0.001** | 0.096 | 0.264 | **0.023** |
| Midbrain PD | Pearson Correlation | 0.379 | **.431*** | 0.379 | 0.353 | 0.377 | 0.295 |
|  | Sig. (2-tailed) | 0.056 | **0.028** | 0.056 | 0.099 | 0.076 | 0.172 |
| **. Correlation is significant at the 0.01 level (2-tailed). | | | | | | | |
| *. Correlation is significant at the 0.05 level (2-tailed). | | | | | | | |

**Table 7:** Correlation between measured imaging parameters and clinical outcome parameters for the group of severe IVH (IVH III & IV/PVHI).
